# Pathophysiological Changes in the Hemostatic System and Antithrombotic Management in Kidney Transplant Recipients

**DOI:** 10.1097/TP.0000000000004452

**Published:** 2023-05-23

**Authors:** Tamar A.J. van den Berg, Gertrude J. Nieuwenhuijs-Moeke, Ton Lisman, Cyril Moers, Stephan J.L. Bakker, Robert A. Pol

**Affiliations:** 1 Department of Surgery, University of Groningen, University Medical Center Groningen, the Netherlands.; 2 Surgical Research Laboratory, Department of Surgery, University of Groningen, University Medical Center Groningen, Groningen, the Netherlands.; 3 Department of Anesthesiology, University of Groningen, University Medical Center Groningen, Groningen, the Netherlands.; 4 Division of Nephrology, Department of Internal Medicine, University of Groningen, University Medical Center Groningen, Groningen, the Netherlands.

## Abstract

Nowadays, the main cause for early graft loss is renal graft thrombosis because kidney transplant outcomes have improved drastically owing to advances in immunological techniques and immunosuppression. However, data regarding the efficacy of antithrombotic therapy in the prevention of renal graft thrombosis are scarce. Adequate antithrombotic management requires a good understanding of the pathophysiological changes in the hemostatic system in patients with end-stage kidney disease (ESKD). Specifically, ESKD and dialysis disrupt the fine balance between pro- and anticoagulation in the body, and further changes in the hemostatic system occur during kidney transplantation. Consequently, kidney transplant recipients paradoxically are at risk for both thrombosis and bleeding. This overview focuses on the pathophysiological changes in hemostasis in ESKD and kidney transplantation and provides a comprehensive summary of the current evidence for antithrombotic management in (adult) kidney transplant recipients.

## INTRODUCTION

Nowadays, the most prominent cause of early graft loss is renal graft thrombosis (RGT). Especially because immunological reasons for early graft loss have virtually vanished because advances in immunological techniques and immunosuppression. RGT, which predominantly originates from renal vein thrombosis, occurs in approximately 1% of transplanted grafts (0.1%–0.3% in case of renal artery thrombosis) and usually results in a transplantectomy.^1,2^ Historically, patients with end-stage kidney disease (ESKD) were considered hypocoagulable with an increased bleeding tendency. In the early 80s, it was shown that the degree of anemia significantly contributed to bleeding time, and the subsequent introduction of recombinant erythropoietin and correction of anemia with hematocrit above 30%, resulted in significantly reduced bleeding time and severity of bleeding complications.^3^

More recently, several studies reported that chronic kidney disease (CKD) itself is a risk factor for thromboembolic events. Paradoxically, patients with ESKD (CKD stage 5) also show an increased bleeding tendency, as may be reflected by a 4% incidence of significant bleeding posttransplantation compared with 1% in general surgery,^1,4^ which is potentially due to profound pro- and anticoagulant changes in ESKD patients. Becuase of these alterations in the hemostatic system and the many confounding factors, patients with CKD/ESKD are often not included in clinical trials investigating the effect of anticoagulant therapy, which complicates decision-making for kidney transplant professionals.^5^ As a result, there are little to no reliable data for formulating evidence-based recommendations regarding perioperative antithrombotic therapy.^6^ To better understand the problems regarding antithrombotic management in kidney transplant recipients, this overview focuses on the pathophysiology of thrombosis and bleeding in ESKD and kidney transplantation and provides a comprehensive summary of the current evidence on antithrombotic management in (adult) kidney transplant recipients.

## GENERAL PHYSIOLOGY OF THE HEMOSTATIC SYSTEM

### Platelet Activation

**FIGURE 1.**
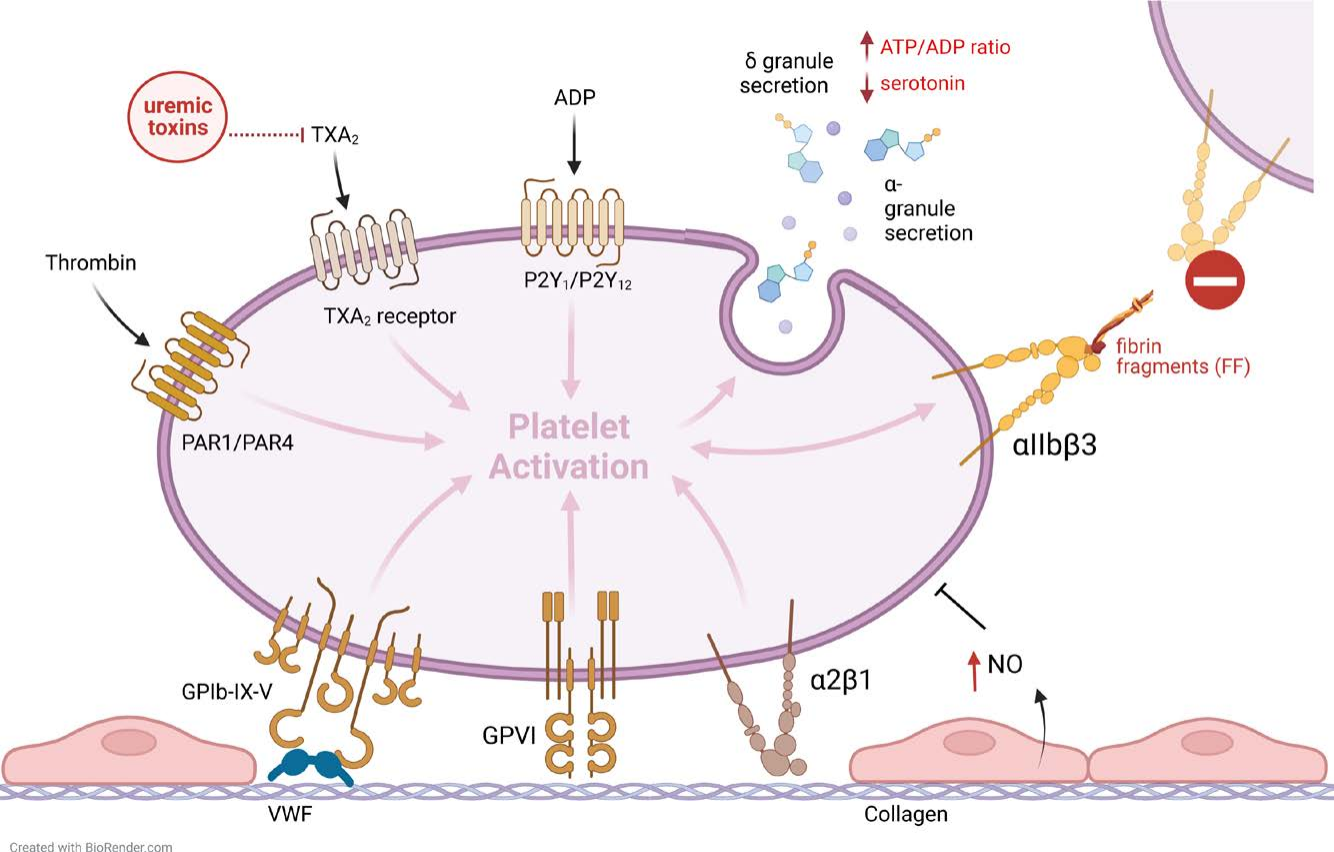
Platelet activation and disturbances due to ESKD and dialysis. ADP, adenosine diphosphate; ATP, adenosine triphosphate; ESKD, end-stage kidney disease; NO, nitric oxide; PAR, protease-activated receptor; TXA_2_, thromboxane 2; VWF, von Willebrand factor.

Platelets are the first responders in cases of acute inflammatory or hemostatic events. Under normal conditions, the endothelium expresses an antithrombotic phenotype. Negative signaling, for example, via nitric oxide (NO), counters the activation and adhesion of platelets near the endothelium.^7,8^ Trauma or inflammation can interfere with the integrity of the endothelium and cause activation of endothelial cells and exposure of the subendothelial matrix, resulting in platelet adhesion, activation, and aggregation (Figure 1).^9^ Activated platelets exocytose α-granules (containing, among others, platelet activating factor, platelet factor 4, von Willebrand factor [VWF], and stored proteins taken up from plasma) and dense (δ-) granules (adenosine triphosphate, adenosine diphosphate (ADP), and serotonin).^10^ Platelets also synthesize and release thromboxane A2 (TXA2), which is a vasoconstrictor and a secondary platelet activating agent.

### Procoagulant Mechanisms

**FIGURE 2.**
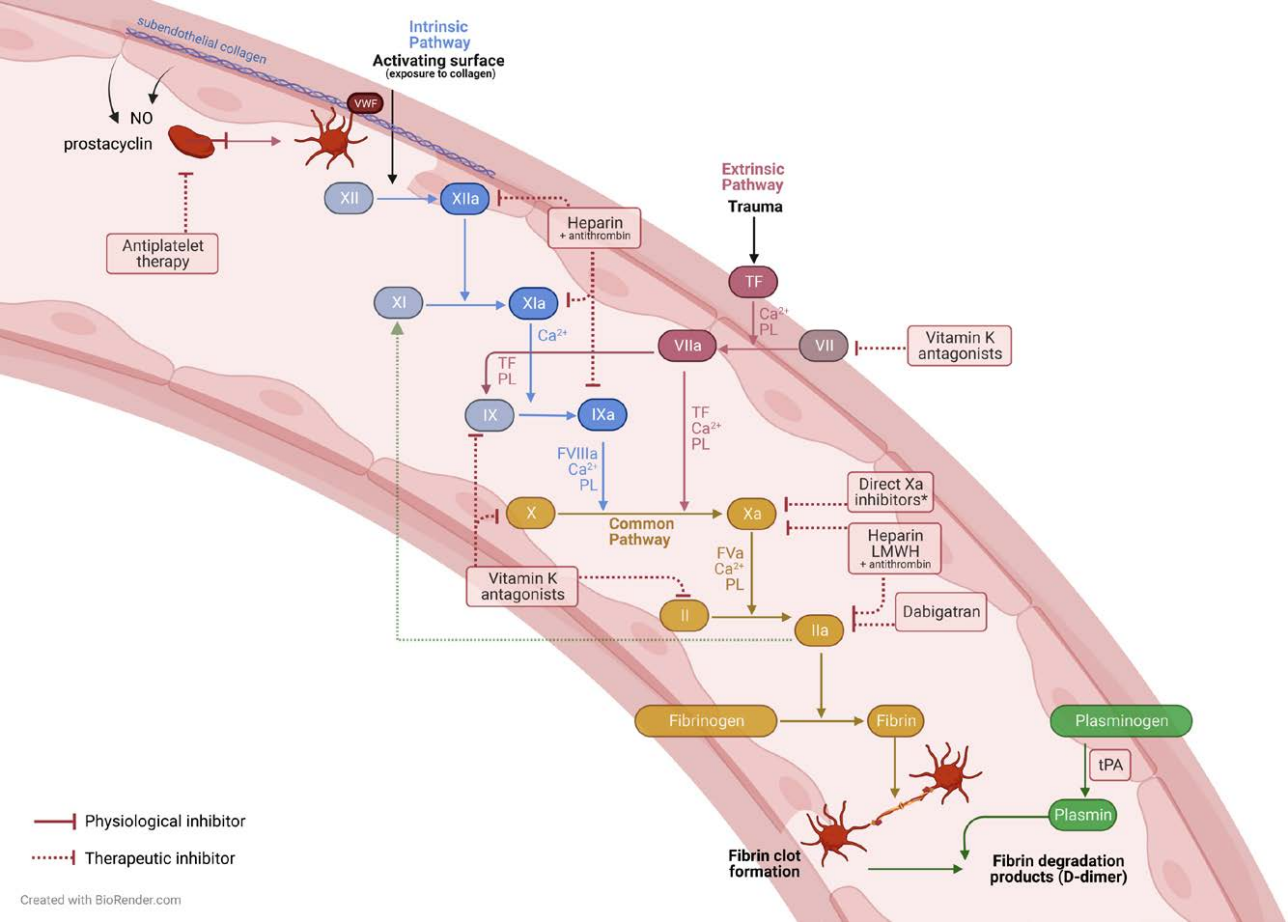
The process of clot formation and the coagulation cascade with sites of inhibition of antiplatelet and anticoagulant drugs. LMWH, low-molecular weight heparin; NO, nitric oxide; PL, phospholipids; TF, tissue factor; tPA, tissue plasminogen activator; VWF, von Willebrand factor.

Upon injury of the endothelium, subendothelial tissue factor (TF) initiates coagulation by complex formation with coagulation factor VIIa. Coagulation factors circulate in the blood in their inactive zymogen form and become activated during coagulation via a catalytic chain reaction. The TF-FVIIa complex activates FX or FIX, eventually leading to the formation of thrombin. Thrombin cleaves fibrinogen to fibrin, which is cross-linked by FXIIIa.^11^ Concomitant amplification of thrombin generation via the intrinsic pathway is established by thrombin activating FXI (Figure 2). Under certain pathological conditions, coagulation may also be activated by the contact activation system (also referred to as the intrinsic pathway of coagulation). Contact activation results in the generation of factor XIIa, which activates FXI (Figure 2).

### Anticoagulant Mechanisms

Various serine protease inhibitors (serpins), such as antithrombin and activated protein C, regulate the coagulation cascade by either direct inhibition of coagulation factors (antithrombin) or inactivation of nonenzymatic cofactors involved in coagulation (activated protein C).

Fibrinolysis is induced when the zymogen plasminogen is activated by an endothelial tissue plasminogen activator (t-PA) and converts plasminogen to plasmin, which cleaves fibrin into degradation products, such as D-dimers. Fibrinolysis is also regulated by inhibitory proteins such as plasminogen activator inhibitor type 1 (PAI-1) and antiplasmin.

## PATHOPHYSIOLOGICAL CHANGES IN HEMOSTASIS IN ESKD

### Nondialysis-dependent CKD Stage 5

ESKD has a profound influence on hemostasis. Alone or in combination with renal replacement therapy, it disrupts the fine balance between procoagulation and anticoagulation mechanisms in the body, resulting in a bleeding tendency on one side and increased risk for thrombosis on the other. Because of reduced kidney filtration, pathophysiological changes in uremic patients include the accumulation of urea, which induces the production of mitochondrial reactive oxygen species, causing endothelial dysfunction,^12,13^ and the accumulation of uremic toxins, of which several have been shown to interfere with platelet function, by, for example, inhibiting TXA2 synthesis.^13^ Furthermore, platelet δ-granules have a decreased ADP and serotonin content, and platelet-vessel wall interactions are impaired, because a decreased amount of GPIb receptors and increased NO production.^7,9,14^

Circulating fibrin degradation products,^15^ which are present in uremic patients, competitively bind to αIIbβ3, impairing platelet-to-platelet interaction. Anemia, which is frequent in ESKD patients, increases the risk of bleeding because of diminished platelet function caused by reduced vessel–wall interaction (as a result of a decreased number of erythrocytes pushing platelets towards the vessel wall), an impaired release of ADP stimulated by erythrocytes, and less scavenging of NO by hemoglobin.^3,16^ Higher VWF levels in uremic patients compensate the relative adhesion defect to some extent,^17^ but altogether, these interactions result in thrombocytopathy and a subsequently impaired primary hemostasis. Additionally, it is important to note that, on top of these changes in platelet function, many ESKD patients experience volume overload associated with hypertension and cardiovascular disease and use antiplatelet therapy indicated for their primary disease or other (cardiovascular) comorbidities, which increase the risk of bleeding. On the other hand, increased levels of circulating fibrinogen and reduced fibrinolytic properties, caused by activation of the renin-angiotensin-aldosterone system, and endothelial cell damage, steer toward hypercoagulability.^9^ An extensive summary of the differential effects of renal failure and dialysis on hemostasis is presented in Table 1.

**TABLE 1.** Differential effects of ESKD and dialysis on the coagulation system (modified from Nieuwenhuijs-Moeke^18^)

Procoagulant	Anticoagulant
**Coagulation cascade** ↑ fibrinogen level, associated with ↑ level of proinflammatory markers (eg, CRP, IL-6) ↑ TF level ↑ FXIIa and FVIIa, ↓ATIII • Activation RAAS, associated with ↑ fibrinogen, ↑ D-dimer, ↑ PAI-1 • PD: ↑ PAI-1, ↑ levels FII and FVII up to FXII	**Coagulation cascade**
**Platelets** • PD: ↑ activity and ↑ count ↑ phosphatidylserine (PS) exposure^19^ due to ↑ platelet activation, which stimulates binding of vit K-dependent coagulation factors. ↑ p-selectin expression, activation of αIIbβ3 facilitating platelet–leucocyte interaction. Results in platelet activation, ROS formation, and increased thrombotic state • Elevated plasma levels of VWF^17^	**Platelets** • Uremic toxins: ↓ TXA2→ ↓adhesion, ↓ aggregation, reversed by HD • ↓ function, disturbance α-granules: ↑ ATP/ADP ratio, ↓ serotonin content ↑ Ca content, with disturbed intracellular flux ↑ ATP release • Competitive binding of FDP bind to αIIbβ3→ ↓adhesion, ↓ aggregation • Reduced collagen-induced platelet aggregation^20^ • Continuous activation dialysis membrane (HD)
**Endothelium** • Endothelial injury through hypoxia, thrombin, oxidants IL-1, TNF-α, IFN-γ, endotoxins… → loss of antithrombotic properties • Production of endothelial microparticles with surface exposure of PS, facilitating thrombin formation and TF, release soluble TF. MP also produced by other cells. • Homocysteine: inhibits thrombomodulin-dependant APC system → permanent activation of thrombin. Interferes with t-PA release→ hypofibrinolysis	**Platelet-vessel wall interaction** • ↓ GPIb platelet receptors • Competitive binding of uremic toxin to αIIbβ3 → reduced binding to fibrinogen • Functional defect VWF-platelet interaction related to uremic toxins • Inhibition platelet aggregation by vasoactive substances: ↑NO production by platelets • ↓ in anemia (less scavenging of NO by haemoglobin)
**Antibodies** • Antiphospholipid antibodies: detectable in many HD patients, significance not clear^21^ • Increased ↑ prevalence APC/antiprotein S antibodies in HD patients with vascular access thrombosis, significance not clear	**Medication** • Platelet aggregation inhibitors • Accumulation of LMWH, FXa inhibitors, thrombin inhibitors

ADP, adenosine diphosphate; APC, activated protein C; ATP, adenosine triphosphate; CRP, C-reactive protein; FDP, fibrin degradation products; GP, glycoprotein; HD, hemodialysis; IFN, interferon factor; IL-6, interleukin-6; LMWH, low-molecular-weight heparin; MP, microparticles; NO, nitric oxide;; PAI, plasminogen activator inhibitor; PD, peritoneal dialysis; PS, phosphatidylserine; RAAS, renin-angiotensin-aldosterone system; ROS, reactive oxygen species; TF, tissue factor; TXA2, thromboxane 2; TNF, tissue necrosis; t-PA, tissue plasminogen activator; VWF, von Willebrand factor.

### Dialysis-dependent CKD Stage 5

Hemodialysis has been shown to affect hemostasis in 3 ways: it leads to (1) thrombocytopathy due to low-grade platelet activation resulting in release of α- and δ-granules following the passing through the dialysis membrane of the extracorporeal circuit, leading to “exhausted” platelets;^22^ (2) rebalancing by removing uremic toxins that, for example, inhibit TXA2 synthesis, and by removing fibrin degradation products, which both disrupt platelet activation and thus primary hemostasis;^9,15,23^ and (3) procoagulant changes, such as circulating antiphospholipid antibodies in hemodialysis patients, and platelet activation with increased plasma levels of PAI-1 and several coagulation factors in peritoneal dialysis patients with hypoalbuminemia.^9,24^

Interestingly, all kidney transplant recipients, regardless of dialysis modality, have been shown to have a comparable and procoagulant hemostatic state before transplantation.^25^ A possible explanation for this might lie in the formation of neutrophil extracellular traps (NETs). These web-like structures, containing DNA, histones, and antimicrobial proteins, are expelled from neutrophils undergoing NETosis and are usually recruited in case of microorganism invasion. However, excessive formation of NETs has been described in CKD patients, hemodialysis, ischemia-reperfusion injury (IRI), and thrombosis.^26-28^ Hypothetically, the combined effects of CKD, hemodialysis, and IRI led to a more pronounced prothrombotic state in dialysis-dependent recipients than preemptive recipients.

### Kidney Transplantation

During kidney transplantation, the alterations in pro- and anticoagulant properties of ESKD patients are further amplified. Activation of hemostatic processes as a result of surgical damage, IRI, and hemodilution contribute to the aggravation of the preoperative hemostatic abnormalities and induce endothelial dysfunction.^29^ Together with the obvious stasis/reduced blood flow due to clamping of the vessels, but also the possible torsion or kinking of the vessels, these conditions include all 3 prerequisites for thrombogenesis as recognized in Virchow’s triad: vessel wall/endothelial injury, stasis, and hypercoagulability.^30^ At the same time, vascular anastomoses, with the risk of anastomotic bleeding, together with acquired coagulation abnormalities secondary to hemodilution add to the already existing bleeding diathesis in kidney transplant recipients.^31^

### Coagulation in Immune Response and Rejection

It has been generally accepted that the coagulation system and platelet activation are an integrated part of the innate immune system, which can be activated upon inflammation or trauma, such as IRI or donor-specific antibodies. Accumulation of platelet aggregates in graft rejection biopsies indicated that platelets could be a marker of graft rejection, but more recently, it was also shown that they are active contributors.^32^ Platelets can contribute to graft rejection in several ways: by functioning as mediators for leukocytes and the damaged endothelium, and through glutamate receptor signaling, which accelerates T cell recruitment.^33,34^ Regarding the coagulation system, serpins such as thrombin, which activates proteinase-activated receptor-1, can contribute to a pro-inflammatory response. Furthermore, the coagulation and complement systems are closely intertwined. It has been shown that the complement system, and especially the alternative pathway, is activated in graft rejection and ischemia-reperfusion. Both C3 and C5 of the complement system can become activated through FIXa, FXa, FXIa, thrombin, and plasmin, and C1 can be activated by FXIIa. In turn, the complement system can contribute to a procoagulatory response by activating thrombin via components of the lectin pathway.^33^ For a more comprehensive description on the interplay between coagulation and complement, we refer to an in-depth review from Oikonomopoulou et al.^33^

## PREVENTION AND TREATMENT OF THROMBOSIS IN KIDNEY TRANSPLANTATION

Most thromboembolic and bleeding events occur in the first days after transplantation and are most likely related to the surgical procedure.^1,35^ Over the years, several groups have studied known and unknown risk factors for thrombosis and bleeding in KTx patients compared with general surgery patients.^35-40^ High heterogeneity limits the possibility to perform thorough multivariate and meta-analyses, but several independent risk factors have been reported. These risk factors are given in Table 2. Well-known, nonmodifiable risk factors are the extremes of age and renal atherosclerosis in both donors and recipients, diabetic nephropathy, past VTE events, and hemodynamic instability. Technical difficulty and longer cold ischemia (>24 h) are independent risk factors as well, but surgical difficulty is highly operator-dependent, and long cold ischemia can be actively avoided.^36,37^ Importantly, although CKD cannot be investigated as a potential risk factor in the kidney transplant setting (given the underlying disease of all patients), it was identified as an independent contributor to thrombosis in another setting.^21^

**TABLE 2.** Transplant-specific risk factors

Renal graft thrombosis	Postoperative bleeding
CKD^41^	Use of vitamin K antagonists^1,42^
Identified inherited/acquired thrombophilic factor^21,43^	Postoperative heparin infusion^1,39,44^
Donor age <6 and >60^37,45,46^	Preemptive kidney transplantation^1^
Recipient age <5 and >50^47^	Cardiovascular disease^1^
Peritoneal dialysis^21,40,48,49^	Longer pretransplant dialysis^50^
Atherosclerosis in external iliac, common iliac, or renal artery (both donor and recipient)^37,50^	Lower body mass index^36,51^
Re-transplantation^48^	***Univariate risk factors***
Diabetic nephropathy^38,48,52^	Longer CIT^36^
Hemodynamic instability^38,39^	Deceased donor type^36^
Technical difficulty^37,38^	ECD transplants^36^
***Univariate risk factors***	
Right donor kidney^a^^37^	
Multiple donor arteries^1,37,53^	
CIT >24 h^40,46^	
Higher body mass index^1^	
Acute cytomegalovirus infection^54,55^	

a Conflicting reports. Probably most related to technical difficulty.^38^

CKD, chronic kidney disease; CIT, cold ischemic time; ECD, extended criteria donor.

In addition, studies have described an apparent increased thrombotic risk and protection against bleeding for patients with obesity (BMI >30 kg/m^2^).^1,36,56^ Although BMI probably interacts with other patient characteristics, it is an important factor to include when identifying the patient at risk and for patient education.^51^ Especially considering the global obesity pandemic, patients with a high BMI are increasingly transplanted.^57^ Many European centers use a BMI cutoff of 35 kg/m^2^ and, in the United States, sometimes even 40 kg/m^2^. Informed preoperative consent now often includes postoperative bleeding as one of the main complications, apart from rejection. The results presented here suggest that, in obese patients, more emphasis should be put on the occurrence of RGT rather than postoperative bleeding.

### Inherited/Acquired Thrombophilia

An important risk factor for increased thrombotic risk is thrombophilia. Patients with thrombophilia have an inherited or acquired hypercoagulable state. Thrombophilia includes, among others, factor V Leiden, prothrombin G20210 mutations, protein C, S, or antithrombin III deficiency, activated protein C resistance, and antiphospholipid or anticardiolipin antibodies.^40,58^ In addition, auto-immune disease systemic lupus erythematosus (SLE) is associated with increased risk of vascular events, such as early atherosclerosis and both arterial and venous thrombosis. A vast amount of SLE patients have antiphospholipid antibodies, also called lupus anticoagulant (LA), which increases the risk for vascular events 6 times. LA negative SLE patients have a 10% to 20% risk of vascular events, compared with 40% of LA positive SLE patients.^59^ Furthermore, acquired thrombophilia, such as LA, is common in ESKD patients, and certain predisposed individuals may encounter thrombosis in response to an external factor, such as transplant surgery.^60^ Already, in 1998, patients with thrombophilia were shown to have a 3.5-fold increased risk for 1-y graft loss, related to both thrombosis and vascular rejection, and in 2001, it was shown that prophylactic anticoagulation of patients with hypercoagulable states due to thrombophilia showed a 2.6-fold reduction of the expected graft thrombosis incidence.^60^ Detection of LA is possible through enzyme-linked immunosorbent assays. However, it is still debated whether routine screening for thrombophilia should be implemented. Anticoagulation should be considered in individuals with known thrombophilia, and postoperative monitoring for development of RGT should be done vigilantly.

Antiplatelet and anticoagulant therapies are, in accordance with their function and purpose, known risk factors for the occurrence of bleeding complications. However, reports investigating the actual risks or benefits of continuing prophylactic or therapeutic antithrombotic therapy in KTx patients are scarce. There are no randomized controlled trials that sufficiently assess the efficacy of anticoagulant/antiplatelet therapy in preventing RGT, and retrospective reports are insufficiently powered to draw solid conclusions. Furthermore, different definitions, for example, what is considered significant bleeding, were used, which makes it difficult to compare studies. Current literature is more focused on the (un)safety of antithrombotic drugs, because of the higher incidence of bleeding complications, than the beneficial effects of preventing thrombosis.

### Antiplatelet Therapy

In CKD patients, thromboembolic prevention is mainly managed with acetylsalicylic acid, its derivative carbasalate calcium (best known as Ascal), and P2Y_12_-inhibitors, such as clopidogrel and ticagrelor, but CKD stage 4 of 5 patients are often underrepresented in studies investigating antiplatelet therapies.^7^ Some patients receive dual antiplatelet therapy, in which case most centers stop one of these before transplantation, but single antiplatelet therapy is generally continued.^61^ Hernandez et al reported 6% more bleeding complications in patients on antiplatelet therapy, but emphasized the importance of additional risk factors, such as complicated bench surgery.^62^ Our group analyzed and published a series of 2000 kidney transplant recipients and found no increased bleeding risk when antiplatelet therapy was continued.^1^ Eng et al. also found no effect of preoperative clopidogrel on bleeding risk, although this might be biased by a low number of events (2 of 10 in a total cohort of 327 patients).^50^ Additionally, Grotz et al showed that low-dose acetylsalicylic acid (aspirin) was associated with improved graft function, longer graft survival, and less proteinuria and hematuria.^44^ As mentioned above, continuation of antiplatelet therapy must, however, be weighed against other risk factors, such as preexisting cardiovascular disease or expected high blood loss.^1^

### Anticoagulants

Anticoagulants that are subscribed can be roughly divided into 3 groups: heparins, vitamin K antagonists (VKA, coumarins), and direct oral anticoagulants (divided into direct FXa inhibitors and direct thrombin [FIIa] inhibitors). Heparins (including low-molecular weight) are used by several transplant centers to prevent RGT, whereas other anticoagulant or antiplatelet therapies are often prophylactically used for other indications, but may contribute to the prevention of RGT.^61^

#### Heparins

Heparin is a glycosaminoglycan that is naturally produced in all vertebrates and was discovered in 1916. Its clinical application was developed by Gordon Murray and Charles Best around the late 1930s,^63^ and already, in these early days, Murray investigated its application in preventing RGT.^64^ It now exists in 2 forms: unfractionated heparin (UFH), with a mix of polysaccharide chains, and low-molecular weight heparin (LMWH), existing of shorter polysaccharides.

#### UFH

UFH potentiates antithrombin III to inhibit several coagulation factors: mainly FXa and FIIa but, to a lesser extent, also FXIIa, FXIa, FIXa, and FVIIa (Figure 2). It has a short half-life of approximately 30 min, and although it can be given safely in patients with CKD, ESKD patients with an eGFR <15 mL/min do require a dose reduction of 33%.^65^ It is preferably administered intravenously, and its effects can be reversed quickly and completely by administering protamine sulfate. Clearance is mainly through endothelial cells and the reticuloendothelial system (phagocytic cells) and, to a lesser extent, through renal excretion.^66,67^ Side effects of UFH include bleeding and reduced platelet count. More severe is the occurrence of heparin-induced thrombocytopenia in selected individuals, which actually may result in thrombosis. Its bioavailability is unpredictable because of a selective binding to positively charged surfaces, which requires activated partial thromboplastin time monitoring.

For its use in kidney transplantation, Ng et al^68^ investigated different heparin protocols in the posttransplant period. Although therapeutic use of UFH showed an increased incidence of significant postoperative bleeding, administration of a prophylactic dose (5000 international units [IU]) subcutaneously was not associated with increased bleeding, a finding consistent with our previously mentioned analysis of 2000 KTx recipients, even in combination with preoperative antiplatelet therapy.^1^ Another small retrospective analysis also showed that kidneys from patients treated with 5000 IU UFH intravenously, before reperfusion, had less microthrombi than the no-heparin group.^69^ Although its effectiveness remains unclear, it appears that prophylactic doses of UFH (eg, 5000 IU intraoperatively) can be safely used without increasing the incidence of significant postoperative bleeding.^1,56,68,70^ Interestingly, heparin and other heparinoids have also been shown to inhibit parts of the complement system, which is directly linked to the coagulation system and an important factor for IRI and antibody-mediated rejection.^71,72^ A recent nonhuman primate study showed that targeted inhibition of complement together with heparin significantly reduced delayed graft function after brain death donation.^73^

#### LMWH

LMWHs also inhibit FXa but target thrombin (FIIa) much less efficiently than UFH (Figure 2). LMWHs include, among others, enoxaparin, dalteparin, tinzaparin, and nadroparin and are often used as VTE-prophylaxis, for example, in patients with increased PADUA scores.^73^ The half-life of LMWHs is approximately 4 h; it has a greater bioavailability than UFH, requires less monitoring, and can be prescribed to both inpatients and outpatients. It is renally eliminated, which, in CKD patients, at least requires empirical dose adjustment of enoxaparin because of increased bleeding risk.^74^ The effects of LMWHs can only partly be reversed by protamine, which is more effective at reversing the activity of heparins toward thrombin than toward factor Xa. It is advised to monitor anti-Xa activity when deviating from weight-adjusted dosing.^75^

#### VKAs (Coumarins)

The most famous coumarin, warfarin, was originally developed as a rodenticide in 1948. It was approved for human use in 1954 and quickly complemented heparin and dicoumarol (for which it took too long to reach a therapeutic effect for practical use) in the clinic under the name Coumadin.^75^ It has a half-life between 24 and 58 h. Later, acenocoumarol and fenprocoumon were developed, with a shorter and longer half-life, respectively.^42^ Coumarins inhibit the posttranslational modification that is required to synthesize vitamin K-dependent coagulation factors FIX, FX, FVII, and FII and protein C and protein S (Figure 2). They have a narrow therapeutic window and interact with certain foods and many other drugs, including antiplatelets and corticosteroids.^68,76^ For ESKD patients, the international normalized ratio target should be lowered because reduced kidney function is associated with significantly increased bleeding risk.^77^ Furthermore, it is associated with the progression of vascular calcification through inhibition of vitamin K-dependent matrix gIa protein, which inhibits vascular calcification.^78^ Many kidney transplant recipients are on a VKA-regimen because of cardiovascular comorbidities, and most transplant professionals discontinue its use or bridge with heparin before transplantation, which, in deceased donor KTx, is not always possible from a time perspective.^61^ Whereas Eng et al. found no effect of warfarin on bleeding risk, several other studies show that VKAs are an independent risk factor for severe postoperative bleeding with a nearly 7-fold increased risk.^1,50,56,75^ However, patients on VKA usually have a vital indication, for example, high CHA_2_DS_2_-VASc score, which makes it difficult to adjust the treatment or dosage.

#### Direct Oral Anticoagulants

More recently introduced anticoagulants are direct thrombin and FXa inhibitors. They inhibit serine proteases of the common pathway (Figure 2). For CKD stages 1 to 3, these are safe, but for CKD 4 or 5, results are poor and conflicting.^79^ The half-time of dabigatran, a direct thrombin inhibitor, is severely influenced by renal function because this compound is 85% renally cleared. Therefore, dabigatran is contraindicated in CKD patients. Direct FXa inhibitors (rivaroxaban, edoxaban, and apixaban) have a half-life between 5 and 12 h and are only partially cleared through renal excretion, but it is expected that this half-life is prolonged in severe CKD stages as well.^65^ The effects of rivaroxaban and apixaban can be reversed by administering andexanet alfa, which is a highly expensive antidote with a mean cost varying between $24 000 and $48 400 per patient.^80^

### Surgical Treatment of RGT

RGT can present as sudden anuria, pain in the surgical region (mostly in the case of renal graft vein thrombosis), and decline in renal function. When renal graft vein thrombosis progresses, pain, swelling, and redness can extend to the ipsilateral leg. Most renal graft thromboses result in transplantectomy,^1^ but, when caught early, surgical treatment can be attempted, although success rates are low. There are some reports describing successful surgical thrombectomy, whereas others achieved resolution of the thrombus using intraarterial fibrinolysis with recombinant t-PA or urokinase.^81,82^ Klepanec et al described a case of acute renal graft artery thrombosis successfully treated by rheolytic thrombectomy, using a rapid-lysis technique with an XG Angiojet catheter and catheter-directed t-PA,^83^ which can also be applied to renal graft vein thrombosis.^84^

## MANAGEMENT AND TREATMENT OF POSTOPERATIVE BLEEDING

Postoperative bleeding can manifest in various degrees of severity. It may originate from the anastomoses, the perirenal tissue, or the recipient’s surgical field. In the immediate postoperative period, gross hematuria or small perigraft or wound hematomas frequently occur.^85^ Hematuria usually resolves in a few days and can be managed conservatively or with short-term bladder irrigation and attention to existing antithrombotic therapy.^86^ It has been shown that the Lich-Gregoir technique significantly lowers the prevalence of hematuria requiring intervention compared with the Politano-Leadbetter or U-stitch (1.7% versus 4.1% and 7.2%).^87^ Small hematomas without graft compression are often detected upon ultrasonography, are rarely of clinical significance, and will eventually resolve without intervention.^86^ Significant postoperative bleeding, requiring blood transfusion and/or surgery, occurs in 0.2% to 4.4% of recipients and is associated with a higher risk of graft loss.^1,35,88^ Known risk factors for postoperative bleeding are given in Table 2. Surgical drain placement in high-risk patients, such as recipients with continued VKA therapy or when surgical hemostasis was already difficult, is an efficient way to detect sentinel bleeding before severe bleeding occurs. Daily duplex imaging and control of hemoglobin levels postoperatively also aid in early detection. A recent study also showed the predictive value of the HAS-BLED score in kidney transplant recipients on anticoagulant therapy, which can aid in identifying patients at increased risk for severe bleeding.^56^

Point-of-care viscoelastic hemostatic tests, such as thromboelastography (TEG) or rotational thromboelastometry (ROTEM) can be used intraoperatively to asses bleeding or thrombotic risk, but data are mainly available from cardiac and liver transplantation surgery. Analyses with ROTEM have shown that ESKD patients, and especially those on peritoneal dialysis compared with hemodialysis, have reduced fibrinolytic properties and thrombin generation capacity, which have been demonstrated with standard laboratory studies, as well.^25,89^ In kidney transplantation, a recent study has shown that high dose-tPA TEG measurements were positively correlated with renal function on the first postoperative day, indicating that it might be beneficial to pair kidneys with known microthrombi and recipients with good functioning fibrinolysis.^90^ However, it remains unclear whether TEG could be predictive of adverse outcomes or give directions for a targeted intervention. In cardiac surgery, activated clotting time (ACT) is used to manage the effect of heparin peroperatively; however, this may be unpredictable in kidney transplantation because haemodilution may prolong ACT. Furthermore, it is still discussed whether the available point-of-care devices to measure ACT are reliable and interchangeable.^91,92^

## WHERE DO WE STAND?

Kidney transplant recipients are at risk for both RGT and postoperative bleeding as a result of various hemostatic changes caused by ESKD, dialysis, and the surgical procedure. A recent European survey among kidney transplant specialists concluded that it remains challenging to identify patients at risk and that there is a need for high-quality studies regarding antithrombotic management in kidney transplantation.^61^ Owing to the paucity of such studies, major thrombosis guidelines have to rely on low-grade evidence, resulting in weaker recommendations and subsequently in varying approaches to antithrombotic management, even within individual centers. What we know now is this: either all available anticoagulant drugs are associated with bleeding, or, if not, their effect on thrombosis reduction has not yet been demonstrated, even if they appear safe. However, postoperative bleeding is readily detectable, and several treatment options are available, whereas RGT usually results in graft loss. Nevertheless, kidney transplant recipients are regularly refrained from antithrombotic treatment. Overall, there seems to be evidence to indicate that intraoperative administration of prophylactic doses (eg, 5000 IU) of UFH before reperfusion is safe and can aid in decreasing the thrombotic risk.^1,68-70^ Antiplatelet therapy, including acetylsalicylic acid, carbasalate calcium, dipyridamole, clopidogrel, and ticagrelor, can be continued safely with minimal risk of postoperative bleeding.^1,44,50^ Continuation of VKAs or continued infusion of therapeutic doses of heparin postoperatively result is significantly associated with bleeding risk.^1,50,56,75^

### Future Studies

This overview highlights the complex changes in the hemostatic system of kidney transplant recipients and the deleterious effect of RGT, warranting appropriate antithrombotic therapy. For the near future, a Delphi study, in which kidney transplant experts draft a guideline based on a general consensus, could offer a solution for the center-wide differences in antithrombotic therapy. That is, at least until adequate randomized controlled trials or well-powered retrospective studies are conducted on which robust recommendations can be based. Most recently, the same approach has led to a consensus for hemostatic management in liver transplantation.^93^ In the end, we do not only want to prevent RGT, but also the unwanted activation of the coagulation cascade and connected systems of inflammation and injury, without compromising on safety. It is clear that a one-size-fits-all approach does not apply to the ESKD population. However, we should be cautious of persisting dogmas about the bleeding tendency of patients with impaired renal function because they might benefit from anticoagulant therapy as well. It is evident that changes already occur in the donor, and future studies should focus on discovering which kidneys would benefit from intervention. It could, for example, be essential to pair donor kidney and recipient based on the recipients’ fibrinolytic capability, as proposed by Walker and colleagues.^90^ Furthermore, now that several studies have concluded that it is safe, future studies should focus on the effect of intraoperative heparin on thrombotic risk. Considering the inhibition of parts of the complement system by heparin and other heparinoids, such trials could function as a double-edged sword, and also provide information on incidence of delayed graft function and antibody-mediated rejection.

### New Antithrombotic Drugs

A real solution might be provided by the development of more suitable drugs. Current anticoagulants mainly block proteases of the common pathway and thus affect all 3 stages of the coagulation process: inhibition, propagation, and amplification. As a result, bleeding complications remain a serious side effect. However, coagulation factors in the intrinsic pathway, such as FXI, appear to be key players in thrombosis but have a minimal role in bleeding, and thus, intrinsic coagulation factors are suggested to be suitable targets for effective and relatively safer anticoagulant drugs.^94^

Many research groups work on FXI(a), as evidenced by a large number of patents,^95,96^ and several have already moved on to different stages of clinical trials. For example, the AXIOMATIC-TKA and ANT-005 TKA trials showed superior DVT prevention by FXIa-inhibitor milvexian and anti-FXI antibody abelacimab in patients undergoing total-knee arthroplasty compared with enoxaparin.^97,98^

Several studies investigating the efficacy of FXI(a)-inhibitors Ionis FXI-LRx (NCT04534114), MK-2060 (NCT05027074), and osocimab (NCT03787368 and NCT04523220) in ESKD patients are currently planned or running. In the PACIFIC-AF study, asundexian (BAY2433334) was compared with apixaban in atrial fibrillation patients and showed a reduced bleeding risk in favor of asundexian. Although the differences between groups were small and the study was not powered to report on thrombosis incidence, these results are very promising, especially because 29% of the patients enrolled had CKD.^99^

## References

[1] Berg TAJ Minnee RC Lisman T. Perioperative antithrombotic therapy does not increase the incidence of early postoperative thromboembolic complications and bleeding in kidney transplantation—a retrospective study. Transplant Int. 2019;32:418–430. 10.1111/tri.13387 PMC6850661 30536448

[2] McCarthy JM Yeung CK Keown PA. Late renal-artery thrombosis after transplantation associated with intraoperative abdominopelvic compression. N Eng J Med. 1990;323:1845–1845. 10.1056/NEJM199012273232617 2247129

[3] Livio M Marchesi D Remuzzi G. Uraemic bleeding: role of anaemia and beneficial effect of red cell transfusions. The Lancet. 1982;320:1013–1015. 10.1016/s0140-6736(82)90050-2 6127502

[4] Chahal R Alexander M Yee K. Impact of a risk-stratified thromboprophylaxis protocol on the incidence of postoperative venous thromboembolism and bleeding. Anaesthesia. 2020;75:1028–1038. 32506488 10.1111/anae.15077

[5] Charytan D Kuntz RE. The exclusion of patients with chronic kidney disease from clinical trials in coronary artery disease. Kidney Int. 2006;70:2021–2030. 17051142 10.1038/sj.ki.5001934 PMC2950017

[6] Surianarayanan V Hoather TJ Tingle SJ. Interventions for preventing thrombosis in solid organ transplant recipients. Cochrane Database of Systematic Reviews 2021;3:CD011557.doi:10.1002/14651858.cd011557.pub2 33720396 10.1002/14651858.CD011557.pub2 PMC8094924

[7] Baaten CCFMJ Schröer JR Floege J. Platelet abnormalities in CKD and their implications for antiplatelet therapy. Clin J Am Soc Nephrol. 2022;17:155–170. 34750169 10.2215/CJN.04100321 PMC8763166

[8] Naseem KM Roberts W. Nitric oxide at a glance. Platelets. 2011;22:148–152. 21050056 10.3109/09537104.2010.522629

[9] Lutz J Menke J Sollinger D. Haemostasis in chronic kidney disease. Nephrol Dial Transplant. 2014;29:29–40. 24132242 10.1093/ndt/gft209

[10] Jenne CN Urrutia R Kubes PP. Bridging hemostasis, inflammation, and immunity. Int J Lab Hematol. 2013;35:254–256. 23590652 10.1111/ijlh.12084

[11] Nieuwenhuijs-Moeke GJ. Perioperative Renal Protective Strategies in Kidney Transplantation. 2019.

[12] D’Apolito M Du X Pisanelli D. Urea-induced ROS cause endothelial dysfunction in chronic renal failure. Atherosclerosis. 2015;239:393–400. 25682038 10.1016/j.atherosclerosis.2015.01.034 PMC4361277

[13] Vanholder R Pletinck A Schepers E. Biochemical and clinical impact of organic uremic retention solutes: a comprehensive update. Toxins (Basel). 2018;10:33. 29316724 10.3390/toxins10010033 PMC5793120

[14] Alves de Sá Siqueira M Brunini TMC Rodrigues Pereira N. Increased nitric oxide production in platelets from severe chronic renal failure patients. Can J Physiol Pharmacol. 2011;89:97–102. 21326340 10.1139/y10-111

[15] Thekkedath UR Chirananthavat T Leypoldt JK. Elevated fibrinogen fragment levels in uremic plasma inhibit platelet function and expression of glycoprotein IIb-IIIa. Am J Hematol. 2006;81:915–926. 16917914 10.1002/ajh.20720

[16] Weisel JW Litvinov RI. Red blood cells: the forgotten player in hemostasis and thrombosis. J Thromb Haemost. 2019;17:271–282. 30618125 10.1111/jth.14360 PMC6932746

[17] Zwaginga J Ijsseldijk M Beeser-Visser N. High von Willebrand factor concentration compensates a relative adhesion defect in uremic blood. Blood. 1990;75:1498–1508. 2156581

[18] Bonomini M Dottori S Amoroso L. Increased platelet phosphatidylserine exposure and caspase activation in chronic uremia. J Thromb Haemost. 2004;2:1275–1281. 15304031 10.1111/j.1538-7836.2004.00837.x

[19] Baaten CCFMJ Sternkopf M Henning T. Platelet function in CKD: A systematic review and meta-analysis. J Am Soc Nephrol. 2021;32:1583–1598. 33941607 10.1681/ASN.2020101440 PMC8425648

[20] Grupp C Troche-Polzien I Stock J. Thrombophilic risk factors in hemodialysis: association with early vascular access occlusion and patient survival in long-term follow-up. PLoS One. 2019;14:e0222102. 31539375 10.1371/journal.pone.0222102 PMC6754127

[21] Ocak G Lijfering WM Verduijn M. Risk of venous thrombosis in patients with chronic kidney disease: identification of high-risk groups. J Thromb Haemost. 2013;11:627–633. 23433091 10.1111/jth.12141

[22] Sirolli V Ballone E Di Stante S. Cell activation and cellular-cellular interactions during hemodialysis: effect of dialyzer membrane. Int J Artif Organs. 2002;25:529–537. 12117292 10.1177/039139880202500607

[23] Bonomini M Bordoni E Ciabattini F. Removal of uremic plasma factors using different dialysis modalities. Hemodial Int. 2006;10:S2s2–S2S4. 10.1111/j.1542-4758.2006.01182.x 16441863

[24] Sloand EM Bern MM Kaldany A. Effect on platelet function of hypoalbuminemia in peritoneal dialysis. Thromb Res. 1986;44:419–25. 3798407 10.1016/0049-3848(86)90320-8

[25] Nieuwenhuijs-Moeke GJ van den Berg TAJ Bakker SJL. Preemptively and non-preemptively transplanted patients show a comparable hypercoagulable state prior to kidney transplantation compared to living kidney donors. PLoS One. 2018;13:e0200537. 30011293 10.1371/journal.pone.0200537 PMC6047796

[26] Jansen MPB Emal D Teske GJD. Release of extracellular DNA influences renal ischemia reperfusion injury by platelet activation and formation of neutrophil extracellular traps. Kidney Int. 2017;91:352–364. 27692564 10.1016/j.kint.2016.08.006

[27] Kim JK Lee HW Joo N. Prognostic role of circulating neutrophil extracellular traps levels for long-term mortality in new end-stage renal disease patients. Clin Immunol. 2020;210:108263. 31629808 10.1016/j.clim.2019.108263

[28] Fuchs TA Brill A Duerschmied D. Extracellular DNA traps promote thrombosis. Proc Natl Acad Sci. 2010;107:15880–15885. 20798043 10.1073/pnas.1005743107 PMC2936604

[29] Basile DP Friedrich JL Spahic J. Impaired endothelial proliferation and mesenchymal transition contribute to vascular rarefaction following acute kidney injury. Am J Physiol Renal Physiol. 2011;300:F721–F733. 21123492 10.1152/ajprenal.00546.2010 PMC3064142

[30] Bagot CN Arya R. Virchow and his triad: a question of attribution. Br J Haematol. 2008;143:180–190. 18783400 10.1111/j.1365-2141.2008.07323.x

[31] Ghadimi K Levy JH Welsby IJ. Perioperative management of the bleeding patient. Br J Anaesth. 2016;117:iii18–iii30. 27940453 10.1093/bja/aew358 PMC5155545

[32] Swaim AF Field DJ Fox-Talbot K. Platelets contribute to allograft rejection through glutamate receptor signaling. J Immunol. 2010;185:6999–7006. 20962257 10.4049/jimmunol.1000929 PMC3137241

[33] Oikonomopoulou K Ricklin D Ward PA. Interactions between coagulation and complement—their role in inflammation. Semin Immunopathol. 2012;34:151–165. 21811895 10.1007/s00281-011-0280-x PMC3372068

[34] Kuo HH Fan R Dvorina N. Platelets in early antibody-mediated rejection of renal transplants. J Am Soc Nephrol. 2015;26:855–863. 25145937 10.1681/ASN.2013121289 PMC4378099

[35] Hachem LD Ghanekar A Selzner M. Postoperative surgical-site hemorrhage after kidney transplantation: incidence, risk factors, and outcomes. Transpl Int. 2017;30:474–483. 28120465 10.1111/tri.12926

[36] Keller AK Jorgensen TM Jespersen B. Identification of risk factors for vascular thrombosis may reduce early renal graft loss: a review of recent literature. J Transplant. 2012;2012:1–9. 10.1155/2012/793461 PMC3369524 22701162

[37] Bakir N Sluiter WJ Ploeg RJ. Primary renal graft thrombosis. Nephrol Dial Transplant. 1996;11:140–147. 8649623

[38] Penny MJ Nankivell BJ Disney AP. Renal graft thrombosis. A survey of 134 consecutive cases. Transplantation. 1994;58:565–569. 8091483

[39] McDonald RA Smith JM Stablein D. Pretransplant peritoneal dialysis and graft thrombosis following pediatric kidney transplantation: A NAPRTCS report. Pediatr Transplant. 2003;7:204–208. 12756045 10.1034/j.1399-3046.2003.00075.x

[40] Irish A. Hypercoagulability in renal transplant recipients. Identifying patients at risk of renal allograft thrombosis and evaluating strategies for prevention. Am J Cardiovasc Drugs. 2004;4:139–149. 15134466 10.2165/00129784-200404030-00001

[41] Morales JM Serrano M Martinez-Flores JA. Antiphospholipid syndrome and renal allograft thrombosis. Transplantation. 2019;103:481–486. 30376553 10.1097/TP.0000000000002510

[42] Lim GB. Warfarin: from rat poison to clinical use [Epub ahead of print. December 14, 2017]. Nat Rev Cardiol. doi:10.1038/nrcardio.2017.172 10.1038/nrcardio.2017.172 29238065

[43] Mathis AS Davé N Shah NK. Bleeding and thrombosis in high-risk renal transplantation candidates using heparin. Ann Pharmacother. 2004;38:537–543. 14766999 10.1345/aph.1D510

[44] Grotz W Siebig S Olschewski M. Low-dose aspirin therapy is associated with improved allograft function and prolonged allograft survival after kidney transplantation. Transplantation. 2004;77:1848–1853. 15223902 10.1097/01.tp.0000129407.31494.45

[45] Nagra A Trompeter RS Fernando ON. The effect of heparin on graft thrombosis in pediatric renal allografts. Pediatr Nephrol. 2004;19:531–535. 15022108 10.1007/s00467-004-1458-4

[46] Ahn S Kim MH Jun KW. The incidence and risk factors for deep vein thrombosis after kidney transplantation in Korea: single-center experience. Clin Transplant. 2015;29:1181–1186. 26447458 10.1111/ctr.12648

[47] Ojo AO Hanson JA Wolfe RA. Dialysis modality and the risk of allograft thrombosis in adult renal transplant recipients. Kidney Int. 1999;55:1952–1960. 10231459 10.1046/j.1523-1755.1999.00435.x

[48] Murphy BG Hill CM Middleton D. Increased renal allograft thrombosis in CAPD patients. Nephrol Dial Transplant. 1994;9:1166–1169. 7800219 10.1093/ndt/9.8.1166

[49] Englesbe MJ Punch JD Armstrong DR. Single-center study of technical graft loss in 714 consecutive renal transplants. Transplantation. 2004;78:623–626. 15446325 10.1097/01.tp.0000128623.26590.6d

[50] Eng M Brock G Li X. Perioperative anticoagulation and antiplatelet therapy in renal transplant: is there an increase in bleeding complication? Clin Transplant. 2011;25:292–296. 20529097 10.1111/j.1399-0012.2010.01293.x

[51] Schold JD Augustine JJ Huml AM. Effects of body mass index on kidney transplant outcomes are significantly modified by patient characteristics. Am J Transplant. 2021;21:751–765. 32654372 10.1111/ajt.16196 PMC8905683

[52] Zorgdrager M Krikke C Hofker SH. Multiple renal arteries in kidney transplantation: a systematic review and meta-analysis. Ann Transplant. 2016;21:469–478. 27470979 10.12659/aot.898748

[53] Parajuli S Lockridge JB Langewisch ED. Hypercoagulability in kidney transplant recipients. Transplantation. 2016;100:719–726. 26413991 10.1097/TP.0000000000000887

[54] Kazory A Ducloux D Coaquette A. Cytomegalovirus-associated venous thromboembolism in renal transplant recipients: a report of 7 cases. Transplantation. 2004;77:597–599. 15084941 10.1097/01.tp.0000109779.36669.0d

[55] Verhoef TI Redekop WK Daly AK. Pharmacogenetic-guided dosing of coumarin anticoagulants: algorithms for warfarin, acenocoumarol and phenprocoumon. Br J Clin Pharmacol. 2014;77:626–641. 23919835 10.1111/bcp.12220 PMC3971980

[56] Hau HM Eckert M Laudi S. Predictive value of HAS-BLED Score regarding bleeding events and graft survival following renal transplantation. J Clin Med. 2022;11:4025. 35887788 10.3390/jcm11144025 PMC9319563

[57] WHO Global Health Observatory data repository. Available at: https://apps.who.int/gho/data/view.main.CTRY2450A?lang=en. Accessed May 25, 2022. 10.1080/02763869.2019.1693231 32069199

[58] Morrissey PE Ramirez PJ Gohh RY. Management of thrombophilia in renal transplant patients. Am J Transplant. 2002;2:872–876. 12392294 10.1034/j.1600-6143.2002.20910.x

[59] Unlu O Zuily S Erkan D. The clinical significance of antiphospholipid antibodies in systemic lupus erythematosus. Eur J Rheumatol. 2016;3:75–84. 27708976 10.5152/eurjrheum.2015.0085 PMC5042235

[60] Friedman GS Meier-Kriesche HU Kaplan B. Hypercoagulable states in renal transplant candidates: impact of anticoagulation upon incidence of renal allograft thrombosis. Transplantation. 2001;72:1073–1078. 11579303 10.1097/00007890-200109270-00016

[61] van den Berg TAJ Lisman T Dor FJMF. Antithrombotic management in adult kidney transplantation: a european survey study. Eur Surg Res. 2021 December 6. doi:10.1159/000521327 10.1159/000521327 PMC9808647 34872084

[62] Hernández D Rufino M Armas S. Retrospective analysis of surgical complications following cadaveric kidney transplantation in the modern transplant era. Nephrol Dial Transplant. 2006;21:2908–2915. 16820375 10.1093/ndt/gfl338

[63] Murray GD Best CH. The use of heparin in thrombosis. Ann Surg. 1938;108:163–177. 17857224 10.1097/00000658-193808000-00002 PMC1386890

[64] Murray G Holden R. Transplantation of kidneys, experimentally and in human cases. Am J Surg. 1954;87:508–515. 13138791 10.1016/0002-9610(54)90411-0

[65] Hughes S Szeki I Nash MJ. Anticoagulation in chronic kidney disease patients--the practical aspects. Clin Kidney J. 2014;7:442–449. 25878775 10.1093/ckj/sfu080 PMC4379338

[66] McRae HL Militello L Refaai MA. Updates in anticoagulation therapy monitoring. Biomedicines. 2021;9:262. 33800804 10.3390/biomedicines9030262 PMC8001784

[67] Blann AD Landray MJ Lip GYH. ABC of antithrombotic therapy: an overview of antithrombotic therapy. BMJ. 2002;325:762–765. 12364307 10.1136/bmj.325.7367.762 PMC1124276

[68] Ng JCY Leung M Landsberg D. Evaluation of heparin anticoagulation protocols in post-renal transplant recipients (EHAP-PoRT study). Can J Hosp Pharm. 2016; 49:217–223. doi:10.1109/24.877341 10.4212/cjhp.v69i2.1538 PMC4853178 27168632

[69] van den Berg TAJ van den Heuvel MC Wiersema-Buist J; TransplantLines Investigators. Aggravation of fibrin deposition and microthrombus formation within the graft during kidney transplantation. Sci Rep. 2021;11:18937. 34556708 10.1038/s41598-021-97629-1 PMC8460629

[70] Denize J Defortescu G Guerrot D. Is intraoperative heparin during renal transplantation useful to reduce graft vascular thrombosis? Prog Urol. 2021;31:531–553. 33516612 10.1016/j.purol.2020.12.007

[71] Zaferani A Talsma D Richter MKS. Heparin/heparan sulphate interactions with complement—a possible target for reduction of renal function loss? Nephrol Dial Transplant. 2014;29:515–522. 23880790 10.1093/ndt/gft243

[72] Danobeitia JS Zens TJ Chlebeck PJ. Targeted donor complement blockade after brain death prevents delayed graft function in a nonhuman primate model of kidney transplantation. Am J Transplant. 2020;20:1513–1526. 31922336 10.1111/ajt.15777 PMC7261643

[73] Barbar S Noventa F Rossetto V. A risk assessment model for the identification of hospitalized medical patients at risk for venous thromboembolism: the Padua Prediction Score. J Thromb Haemost. 2010;8:2450–2457. 20738765 10.1111/j.1538-7836.2010.04044.x

[74] Lim W Dentali F Eikelboom JW. Meta-analysis: low-molecular-weight heparin and bleeding in patients with severe renal insufficiency. Ann Intern Med. 2006;144:673–684. 16670137 10.7326/0003-4819-144-9-200605020-00011

[75] Musetti C Quaglia M Cena T. Impact of pre-transplant antiaggregant and anticoagulant therapies on early hemorrhagic and cardiovascular events after kidney transplantation. J Nephrol. 2015;28:757–764. 25743391 10.1007/s40620-015-0185-1

[76] di Minno A Frigerio B Spadarella G. Old and new oral anticoagulants: Food, herbal medicines and drug interactions. Blood Rev. 2017;31:193–203. 28196633 10.1016/j.blre.2017.02.001

[77] Limdi NA Limdi MA Cavallari L. Warfarin dosing in patients with impaired kidney function. Am J Kid Dis. 2010;56:823–831. 20709439 10.1053/j.ajkd.2010.05.023 PMC2963672

[78] El-Abbadi M Giachelli CM. Mechanisms of vascular calcification. Adv Chronic Kidney Dis. 2007;14:54–66. 17200044 10.1053/j.ackd.2006.10.007

[79] Aursulesei V Costache II. Anticoagulation in chronic kidney disease: from guidelines to clinical practice. Clin Cardiol. 2019;42:774–782. 31102275 10.1002/clc.23196 PMC6671778

[80] Fanikos J Goldstein JN Lovelace B. Cost-effectiveness of andexanet alfa versus four-factor prothrombin complex concentrate for the treatment of oral factor Xa inhibitor-related intracranial hemorrhage in the US. J Med Econ. 2022;25:309–320. 35168455 10.1080/13696998.2022.2042106

[81] Rouvière O Berger P Béziat C. Acute thrombosis of renal transplant artery. Transplantation. 2002;73:403–409. 11884937 10.1097/00007890-200202150-00014

[82] Iwami D Harada H Miura M. Successfully rescued renal graft artery thrombosis by ex vivo thrombectomy: a case report. Transplant Proc. 2009;41:1951–1953. 19545764 10.1016/j.transproceed.2009.02.069

[83] Klepanec A Balazs T Bazik R. Pharmacomechanical thrombectomy for treatment of acute transplant renal artery thrombosis. Ann Vasc Surg. 2014;28:1314.e11–1314.e14. 10.1016/j.avsg.2013.09.016 24361385

[84] Harraz AM Shokeir AA Soliman SA. Salvage of grafts with vascular thrombosis during live donor renal allotransplantation: a critical analysis of successful outcome. Int J Urol. 2014;21:999–1004. 24861882 10.1111/iju.12485

[85] Pawlicki J Cierpka L Król R. Risk factors for early hemorrhagic and thrombotic complications after kidney transplantation. Transplant Proc. 2011;43:3013–3017. 21996213 10.1016/j.transproceed.2011.07.018

[86] Rodríguez Faba O Boissier R Budde K. European Association of Urology guidelines on renal transplantation: update 2018. Eur Urol Focus. 2018;4:208–215. 30033070 10.1016/j.euf.2018.07.014

[87] Alberts VP Idu MM Legemate DA. Ureterovesical anastomotic techniques for kidney transplantation: a systematic review and meta-analysis. Transplant Int. 2014;27:593–605. 10.1111/tri.12301 24606191

[88] Dimitroulis D Bokos J Zavos G. Vascular complications in renal transplantation: a single-center experience in 1367 renal transplantations and review of the literature. Transplant Proc. 2009;41:1609–1614. 19545690 10.1016/j.transproceed.2009.02.077

[89] Gäckler A Rohn H Lisman T. Evaluation of hemostasis in patients with end-stage renal disease. PLoS One. 2019;14:e0212237. 30785941 10.1371/journal.pone.0212237 PMC6382154

[90] Walker CB Moore HB Nydam TL. The use of thromboelastography to assess post-operative changes in coagulation and predict graft function in renal transplantation. Am J Surg. 2020;220:1511–1517. 32878689 10.1016/j.amjsurg.2020.08.019 PMC7450953

[91] Dirkmann D Nagy E Britten MW. Point-of-care measurement of activated clotting time for cardiac surgery as measured by the Hemochron signature elite and the Abbott i-STAT: agreement, concordance, and clinical reliability. BMC Anesthesiol. 2019;19:174. 31492108 10.1186/s12871-019-0846-z PMC6728977

[92] Lee JM Park EY Kim KM. Comparison of activated clotting times measured using the Hemochron Jr. Signature and Medtronic ACT Plus during cardiopulmonary bypass with acute normovolemic haemodilution. J Int Med Res. 2018;46:873–882. 28974132 10.1177/0300060517731952 PMC5971518

[93] Montalvá E Rodríguez-Perálvarez M Blasi A; Spanish Society of Liver Transplantation and the Spanish Society of Thrombosis and Haemostasis. Consensus statement on hemostatic management, anticoagulation, and antiplatelet therapy in liver transplantation. Transplantation. 2022;106:1123–1131. 34999660 10.1097/TP.0000000000004014 PMC9128618

[94] van Montfoort ML Meijers JCM. Anticoagulation beyond direct thrombin and factor Xa inhibitors: Indications for targeting the intrinsic pathway? Thromb Haemost. 2013;110:223–232. 23739841 10.1160/TH12-11-0803

[95] Al-Horani RA. Factor XI(a) inhibitors for thrombosis: an updated patent review (2016-present). Expert Opin Ther Pat. 2020;30:39–55. 31847619 10.1080/13543776.2020.1705783 PMC7515655

[96] Afosah DK Ofori E Mottamal M. Factor IX(a) inhibitors: an updated patent review (2003-present). Expert Opin Ther Pat. 2022;32:381–400. 34991418 10.1080/13543776.2022.2026926 PMC8957558

[97] Weitz JI Strony J Ageno W. Milvexian for the prevention of venous thromboembolism. N Eng J Med. 2021;385:2161–2172. 10.1056/NEJMoa2113194 PMC9540352 34780683

[98] Verhamme P Yi BA Segers A. Abelacimab for prevention of venous thromboembolism. N Eng J Med. 2021;385:609–617. 10.1056/NEJMoa2105872 34297496

[99] Piccini JP Caso V Connolly SJ. Safety of the oral factor XIa inhibitor asundexian compared with apixaban in patients with atrial fibrillation (PACIFIC-AF): a multicentre, randomised, double-blind, double-dummy, dose-finding phase 2 study. The Lancet. 2022;399:1383–1390. 10.1016/S0140-6736(22)00456-1 35385695

